# Dynamic Analysis of an Epidemic Model Considering Personal Alert on a Complex Network

**DOI:** 10.3390/e25101437

**Published:** 2023-10-11

**Authors:** Fengling Jia, Ziyu Gu, Lixin Yang

**Affiliations:** 1 School of Mathematics, Chengdu Normal University, Chengdu 611130, China; cdjiafl@163.com; 2 School of Mathematics and Data Science, Shaanxi University of Science & Technology, Xi’an 710021, China; guzy@163.com

**Keywords:** epidemic model, complex networks, individual alertness, quarantine, stability

## Abstract

This paper proposes a SIQRS epidemic model with birth and death on a complex network, considering individual alertness. In particular, we investigate the influence of the individual behavior in the transmission of epidemics and derive the basic reproduction number depending on birth rate, death rate, alertness rate, quarantine rate. In addition, the stabilities of the disease-free equilibrium point and endemic equilibrium point are analyzed via stability theory. It is found that the emergence of individual behavior can influence the process of transmission of epidemics. Our results show that individual alertness rate is negatively correlated with basic reproduction number, while the impact of individual alertness on infectious factor is positively correlated with basic reproduction number. When the basic reproduction number is less than one, the system is stable and the disease is eventually eradicated. Nevertheless, there is an endemic equilibrium point under the condition that the basic reproduction number is more than one. Finally, numerical simulations are carried out to illustrate theoretical results.

## 1. Introduction

As we know, infectious diseases have posed a danger to human physical and mental health over the years. Historically, the epidemics of infectious diseases have wreaked havoc on human existence and caused huge losses to the social economy. In the early days, researchers proposed mathematical models for the spread of epidemics, which provided a theoretical method to study of infectious diseases [1,2]. Hence, variety epidemic models for transmission prediction and control have been proposed to study the mechanisms of epidemic transmission.

Epidemic dynamic transmission models, constructed using differential equations, can describe the dynamic behavior of many epidemics. However, most existing models neglect the variation in individual infectious capacity. In fact, numerous patterns for human contact exist [3]. It is well known that complex networks have various topological structures and this characteristic can well describe the above case. Therefore, it is more realistic to analyze the transmission dynamics of infectious diseases on complex networks. Currently, the network models that more realistically reflect the spread of epidemics in the population are based on the small-word and scale-free network [4,5,6]. Based on the framework of the two network models, scholars have increasingly studied the transmission dynamics of epidemic models [7,8,9,10,11,12,13,14].

Nevertheless, most studies assumed that the number of individuals is a constant, i.e., birth and death are not considered. But the spread of some epidemics can influence population size, such as the birth of individuals leading to the growth of the network and the death leading to the reduction of the network [15,16,17,18,19,20,21,22]. Li et al. [21] proposed a SIRS epidemic model with births and deaths and found that the basic reproduction number was an important factor influencing the dynamics of the network-based SIRS model. Building on the research in Ref. [21], Huang et al. [22] investigated that, under certain conditions, local equilibrium can be shown to be globally attractive using the monotone iterative technique. Birth and death can complicate the degree distribution of the network, making it significantly more difficult to study. Later, Liu et al. [23] considered an empty lattice network, in which birth and death do not affect the degree distribution. They presented an epidemic model that considered birth and death rates on regular and scale-free networks, respectively. Their results showed that there is an epidemic threshold on regular network and no epidemic threshold for scale-free network in the thermodynamic limit. Based on the above network model framework, a large number of epidemic transmission models are studied on the empty lattice network with birth and death [24,25,26,27,28]. N. Masuda and N. Konno [25] analyzed epidemic models with different contact rates on complex network with birth and death and studied the effect of heterogeneity on epidemic threshold. Zhang and Jin [26] derived a transmission threshold $R_{0}$ dependent on birth and death rate and compared the effectiveness of various immunization methods for epidemic transmission. It was shown that targeted and acquaintance immunization strategies were somewhat more effective than proportional immunization. Yuan et al. [27] proposed an SIR model with two susceptible groups and proposed an effective method of controlling the spread of the epidemic. Also, the global asymptotically stability of local equilibrium has been proven in ref. [28]. Although epidemic models have been studied extensively, there are few results concerning individual alertness epidemic models on complex networks. This paper will investigate the dynamics of the epidemic model with individual alertness on an empty lattice network.

The organization of this paper is as follows. In Section 2, considering individual alertness, we construct a SIQRS network epidemic model with birth and death. Then, in Section 3, we prove the existence of a disease-free equilibrium point and an intrinsic equilibrium point. Moreover, the effect of individual alertness behavior on the basic reproduction number is studied. In addition, we study the global stability of the disease-free equilibrium point and the global stability of the endemic equilibrium point. In Section 4, numerical simulations are used to illustrate the results of the theoretical analysis, which show that individual alertness and quarantine behaviors play a significant role in the spreading of epidemics. In Section 5, we take the Coronavirus Disease 2019 as an example to describe the impact of alertness on the spread of infectious diseases, and the conclusions are shown in Section 6.

## 2. The Model

We consider a scale-free network with N nodes, each of which is either empty or occupied by at most one individual, these individuals can be susceptible, infected, quarantined, or recovered and can be represented by *V*, *S*, *I*, *Q*, *R*, respectively. The following variables $V_{k}$, $S_{k}$, $I_{k}$, $Q_{k}$, $R_{k}$ indicate the densities of empty nodes, susceptible, infected, quarantined and recovered individuals with degree of k (1≤k≤M), and satisfy the condition that $V_{k}+S_{k}+I_{k}+Q_{k}+R_{k}=1$. Furthermore, each node in each position will change its state with a certain rate.

**Figure 1:**
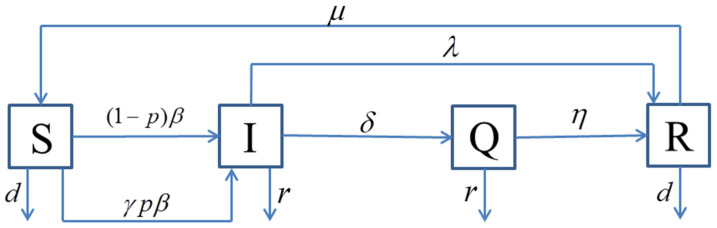
The compartment diagram of SIQRS.

Assuming that individuals are born susceptible, and that birth can only occur at an empty node with the birth rate of b, if an individual has an infected neighbor, the individual produces an alert behavior and responses to avoid contact with the infected individual. The compartment diagram is shown in Figure 1.

Based on the mean field theory [29], our SIQRS epidemic model is described by the differential equation

(1) $$\left\{ \begin{array}{l} \frac{dS_{k}(t)}{dt}=b(1-S_{k}(t)-I_{k}(t)-Q_{k}(t)-R_{k}(t))-[(1-p)\beta +\gamma p\beta ]kS_{k}(t)\theta (t)+\mu R_{k}(t)-dS_{k}(t),\\ \frac{dI_{k}(t)}{dt}=[(1-p)\beta +\gamma p\beta ]kS_{k}(t)\theta (t)-(\lambda +\delta +r)I_{k}(t),\\ \frac{dQ_{k}(t)}{dt}=\delta I_{k}(t)-(r+\eta )Q_{k}(t),\\ \frac{dR_{k}(t)}{dt}=\lambda I_{k}(t)+\eta Q_{k}(t)-(\mu +d)R_{k}(t). \end{array}\right.$$

where p denotes the rate of an individual taking protective measures. In other words, if a susceptible individual has an infected neighborhood, the individual will take an alert behavioral reactivity to avoid contact with other infected individuals, and β is the rate that a susceptible individual will be infected via contacting and γβ represents the rate of an alert individual being infected. If an individual dies, there is an empty node. d is the natural death rate, α is the death rate due to disease, r=d+α>d, δ is the probability that infected individuals become quarantined through the quarantine measure, λ and η are the recovery rate of infected individuals and quarantined individuals, respectively, and μ is the rate that recovered individuals revert to susceptible. All the above parameters are non-negative.

For simplicity, we assume that the degrees of the interconnected nodes in the network are uncorrelated. For the network with uncorrelated degrees, θ(t) is the probability that a randomly chosen link emanating from a node of degree k leads to a node of degree j, which can be described as

$$\theta (t)=\frac{\sum_{j}jp(j)I_{j}(t)}{\langle k\rangle }.$$

where $\langle k\rangle =\sum_{k}kp(k)$ is the average degree of the network.

In order to obtain the positive invariant set of system (1), we sum the four equations of the system (1).

$$\begin{array}{cc} \frac{d(S_{k}(t)+I_{k}(t)+Q_{k}(t)+R_{k}(t))}{dt} & =b(1-S_{k}(t)-I_{k}(t)-Q_{k}(t)-R_{k}(t))-dS_{k}(t)-rI_{k}(t)-rQ_{k}(t)-dR_{k}(t)\\ & \leq b-b(S_{k}(t)+I_{k}(t)+Q_{k}(t)+R_{k}(t))-d(S_{k}(t)+I_{k}(t)+Q_{k}(t)+R_{k}(t)). \end{array}$$

Hence $\underset{t\to \infty }{\lim}\sup(S_{k}(t)+I_{k}(t)+Q_{k}(t)+R_{k}(t))\leq \frac{b}{b+d}$. The positive invariant set of system (1) is

$$\Gamma =\left\{(S_{1},I_{1},Q_{1},R_{1},\cdots ,S_{M},I_{M},Q_{M},R_{M})\in R^{4M},S_{k}+I_{k}+Q_{k}+R_{k}\leq \frac{b}{b+d}\right\}.$$

## 3. Stability Analysis

In this section, we focus on the existence of equilibria and their stability of system (1).

### 3.1. The Existence of Equilibrium

Let $\left(S_{k}^{*},I_{k}^{*},Q_{k}^{*},R_{k}^{*}\right)$ be the positive equilibrium of mode (1). By setting the right-hand side of system (1) to be zero, one can obtain

(2) $$\left\{ \begin{array}{l} b(1-S_{k}^{*}-I_{k}^{*}-Q_{k}^{*}-R_{k}^{*})-[(1-p)\beta +\gamma p\beta ]kS_{k}^{*}\theta^{*}+\mu R_{k}^{*}-dS_{k}^{*}=0,\\ {}[(1-p)\beta +\gamma p\beta ]kS_{k}^{*}\theta^{*}-(\lambda +\delta +r)I_{k}^{*}=0,\\ \delta I_{k}^{*}-(r+\eta )Q_{k}^{*}=0,\\ \lambda I_{k}^{*}+\eta Q_{k}^{*}-(\mu +d)R_{k}^{*}=0. \end{array}\right.$$

We solve the system of Equation (2) and obtain

(3) $$I_{k}^{*}=\frac{[(1-p)\beta +\gamma p\beta ]kS_{k}^{*}\theta^{*}}{\lambda +\delta +r}.$$

(4) $$Q_{k}^{*}=\frac{\delta [(1-p)\beta +\gamma p\beta ]kS_{k}^{*}\theta^{*}}{\left(r+\eta )(\lambda +\delta +r\right)}.$$

(5) $$R_{k}^{*}=\frac{(\delta \eta +\lambda r+\lambda \eta )[(1-p)\beta +\gamma p\beta ]kS_{k}^{*}\theta^{*}}{\left(r+\eta )(\mu +d)(\lambda +\delta +r\right)}.$$

For simplicity, we define the new variable $\tilde{\beta }=(1-p+p\gamma )\beta$. Then, substitute the above Equations (3)–(5) into the first equation of (2); this substitution yields the solution (6).

(6) $$S_{k}^{*}=\frac{b(r+\eta )(\mu +d)(\lambda +\delta +r)}{(b+d)(r+\eta )(\mu +d)(\lambda +\delta +r)+[(b+r)(\mu +d)(r+\eta +\delta )+(b+d)(\delta \eta +\lambda r+\lambda \eta )]\tilde{\beta }k\theta^{*}}.\;I_{k}^{*}=\frac{b(r+\eta )(\mu +d)[(1-p)\beta +\gamma p\beta ]k\theta^{*}}{(b+d)(r+\eta )(\mu +d)(\lambda +\delta +r)+[(b+r)(\mu +d)(r+\eta +\delta )+(b+d)(\delta \eta +\lambda r+\lambda \eta )]\tilde{\beta }k\theta^{*}}.\;Q_{k}^{*}=\frac{b\delta (\mu +d)[(1-p)\beta +\gamma p\beta ]k\theta^{*}}{(b+d)(r+\eta )(\mu +d)(\lambda +\delta +r)+[(b+r)(\mu +d)(r+\eta +\delta )+(b+d)(\delta \eta +\lambda r+\lambda \eta )]\tilde{\beta }k\theta^{*}}.\;R_{k}^{*}=\frac{b(\delta \eta +\lambda r+\lambda \eta )[(1-p)\beta +\gamma p\beta ]k\theta^{*}}{(b+d)(r+\eta )(\mu +d)(\lambda +\delta +r)+[(b+r)(\mu +d)(r+\eta +\delta )+(b+d)(\delta \eta +\lambda r+\lambda \eta )]\tilde{\beta }k\theta^{*}}.$$

From Equation (6), a self-consistency equation can be obtained.

(7) $$\begin{array}{cc} \theta = & \frac{\sum_{k}kp(k)I_{k}^{*}}{\langle k\rangle }\\ = & \frac{b(r+\eta )(\mu +d)[(1-p)\beta +\gamma p\beta ]\sum_{k}k^{2}p(k)\theta }{\langle k\rangle ((b+d)(r+\eta )(\mu +d)(\lambda +\delta +r)+[(b+r)(\mu +d)(r+\eta +\delta )+(b+d)(\delta \eta +\lambda r+\lambda \eta )][(1-p)\beta +\gamma p\beta ]k\theta }\\ = & \frac{b(r+\eta )(\mu +d)}{\langle k\rangle [(b+r)(\mu +d)(r+\eta +\delta )+(b+d)(\delta \eta +\lambda r+\lambda \eta )]}\times \\ & \sum_{k}\frac{[(b+r)(\mu +d)(r+\eta +\delta )+(b+d)(\delta \eta +\lambda r+\lambda \eta )][(1-p)\beta +\gamma p\beta ]k^{2}p(k)\theta }{(b+d)(r+\eta )(\mu +d)(\lambda +\delta +r)+[(b+r)(\mu +d)(r+\eta +\delta )+(b+d)(\delta \eta +\lambda r+\lambda \eta )][(1-p)\beta +\gamma p\beta ]k\theta }\\ = & \frac{b(r+\eta )(\mu +d)}{\langle k\rangle [(b+r)(\mu +d)(r+\eta +\delta )+(b+d)(\delta \eta +\lambda r+\lambda \eta )]}\cdot \sum_{k}\frac{\beta^{\prime }k^{2}p(k)\theta }{1+\beta^{\prime }k\theta }\\ = & f(\theta ). \end{array}$$

For simplicity, we define the new variables

A=[(b+r)(μ+d)(r+η+δ)+(b+d)(δη+λr+λη)] B=[(1−p)β+γpβ],C=(b+d)(r+η)(μ+d)(λ+δ+r)

where $\beta^{\prime }=\frac{AB}{C}$, obviously θ=0 is a solution of Equation (7), and

$$\begin{array}{cc} f(1) & =\frac{b(r+\eta )(\mu +d)}{A}\cdot \sum_{k}\frac{\beta^{\prime }k^{2}p(k)}{1+\beta^{\prime }k}\\ & <\frac{b(r+\eta )(\mu +d)}{A}<1 \end{array}$$

The conditions for the existence of positive solutions of Equation (7) are given below. In order to have solutions θ(θ∈(0,1]), we need the condition $f^{\prime }(\theta )|{}_{\theta =0}>1$

$$f^{\prime }(\theta )=\frac{b(r+\eta )(\mu +d)}{\langle k\rangle A}\sum_{k}\frac{\beta^{\prime }k^{2}p(k)}{{\left(1+\beta^{\prime }k\theta \right)}^{2}}.$$

$$f^{\prime }(\theta )|{}_{\theta =0}=\frac{b[(1-p)\beta +\gamma p\beta ]}{\left(b+d)(\lambda +\delta +r\right)}\cdot \frac{\langle k^{2}\rangle }{\langle k\rangle }>1.$$

where $\langle k^{2}\rangle =\sum_{k}k^{2}p(k)$ is the second moment of the degree distribution. Therefore, we define the basic reproduction number, which can be obtained as follows:

$$R_{0}=\frac{b[(1-p)\beta +\gamma p\beta ]}{\left(b+d)(\lambda +\delta +r\right)}\cdot \frac{\langle k^{2}\rangle }{\langle k\rangle }.$$

In what follows, we study the relationship between the basic reproduction number $R_{0}$ and individual alertness p, individual alertness to infectious factor γ. Based on the description of the basic reproduction number, we notice that $R_{0}=const.\tilde{\beta }$; hence, it is a linear function of both p and γ, in particular,

$$\frac{d}{dp}R_{0}=const.\tilde{\beta }$$

which is negative if γ<1. Similarly

$$\frac{d}{d\gamma }R_{0}=const.pb>0.$$

In summary, if $R_{0}<1$, the system has a disease-free equilibrium point $E_{0}$ on the boundary of Γ.

$$E_{0}(\frac{b}{b+d},0,0,0,\cdots ,\frac{b}{b+d},0,0,0).$$

If $R_{0}>1$, the system has an endemic equilibrium $E^{*}$ in the interior of Γ.

$$E^{*}(S_{1}^{*},I_{1}^{*},Q_{1}^{*},\mathrm{and}R_{1}^{*},\cdots ,S_{M}^{*},I_{M}^{*},Q_{M}^{*},R_{M}^{*}).$$

### 3.2. Stability of the Equilibria

In this subsection, we focus first on the local stability of the disease-free equilibrium point $E_{0}$, the endemic equilibrium point $E^{*}$ by using the linear approximation method and the global stability of the endemic equilibrium point $E_{0}$ by using the Lyapunov function of the system (1). The main results are presented as the following theorems.

**Theorem** **1.** *If*  $R_{0}<1$*, the disease-free equilibrium* $E_{0}$ *of the epidemic model (1) is locally asymptotically stable. Otherwise, the disease-free equilibrium point* $E_{0}$ *of the epidemic model (1) is unstable, and the epidemic model (1) has a unique epidemic equilibrium* $E^{*}$.

**Proof.**  The Jacobian matrix at the disease-free equilibrium $E^{0}$ is

$$J(E_{0})=\left(\begin{array}{ccccc} J_{11} & & J_{1j} & & J_{1M}\\ \vdots & \ddots & & & \vdots \\ 0 & & J_{jj} & & J_{jM}\\ \vdots & & & \ddots & \vdots \\ J_{M1} & & J_{Mj} & & J_{MM} \end{array}\right).$$

where $J_{11}=\left(\begin{array}{cccc} -(b+d) & -b & -b & -b+\mu \\ 0 & -(\lambda +\delta +r) & 0 & 0\\ 0 & \delta & -(r+\eta ) & 0\\ 0 & \lambda & \eta & -(\mu +d) \end{array}\right)$, $J_{jj}=\left(\begin{array}{cccc} -(b+d) & -b-h_{jj} & -b & -b+\mu \\ 0 & -(\lambda +\delta +r)+h_{jj} & 0 & 0\\ 0 & \delta & -(r+\eta ) & 0\\ 0 & \lambda & \eta & -(\mu +d) \end{array}\right)$,$J_{ij}=\left(\begin{array}{cccc} 0 & -h_{ij} & 0 & 0\\ 0 & h_{ij} & 0 & 0\\ 0 & 0 & 0 & 0\\ 0 & 0 & 0 & 0 \end{array}\right)$, $h_{ij}=[(1-p)\beta +\gamma p\beta ]iS_{i}^{*}g(j)$ and $g(j)=\frac{jp(j)}{\langle k\rangle }$, i,j=1,2,⋯,M. The structure of the matrix $J(E_{0})$ implies that it has M eigenvalues equal to −(b+d), M eigenvalues equal to −(r+η), and M eigenvalues equal to −(μ+d). The 3M + 1-th eigenvalue is −(λ+δ+r), and the remaining M − 1 eigenvalues are eigenvalues of the matrix, where

$$F=\left(\begin{array}{cccccc} h_{22}-(\lambda +\delta +r) & h_{23} & \cdots & h_{2j} & \cdots & h_{2M}\\ h_{32} & h_{33}-(\lambda +\delta +r) & \cdots & h_{3j} & \cdots & h_{3M}\\ \vdots & \vdots & & \vdots & & \vdots \\ h_{j2} & h_{j3} & \cdots & h_{jj}-(\lambda +\delta +r) & \cdots & h_{jM}\\ \vdots & \vdots & & \vdots & & \vdots \\ h_{M2} & h_{M3} & \cdots & h_{Mj} & \cdots & h_{MM}-(\lambda +\delta +r) \end{array}\right).$$

We perform a row–column similarity transformation on F to obtain the transformed matrix

$$F^{*}=\left(\begin{array}{cccccc} -(\lambda +\delta +r) & 0 & \cdots & 0 & \cdots & h_{2M}\\ 0 & -(\lambda +\delta +r) & \cdots & 0 & \cdots & (h_{22}+h_{33})\frac{g(M)}{g(3)}\\ \vdots & \vdots & & \vdots & & \vdots \\ 0 & 0 & \cdots & -(\lambda +\delta +r) & \cdots & \sum_{i=2}^{j}h_{ii}\frac{g(M)}{g(j)}\\ \vdots & \vdots & & \vdots & & \vdots \\ 0 & 0 & \cdots & 0 & \cdots & \sum_{i=2}^{M}h_{ii}-(\lambda +\delta +r) \end{array}\right).$$

Thus, the matrix $J(E_{0})$ has M − 2 eigenvalues equal to −(λ+δ+r). The 4M-th eigenvalue is

(8) $$\sum_{i=2}^{M}h_{ii}-(\lambda +\delta +r)=\sum_{i=2}^{M}[(1-p)\beta +\gamma p\beta ]iS_{i}\frac{ip(i)}{\langle k\rangle }-(\lambda +\delta +r)=(\lambda +\delta +r)(R_{0}-1).$$

And it follows that

(9) $$(\lambda +\delta +r)(R_{0}-1)<0$$

if and only if $R_{0}<1$. In this case, all the eigenvalues of $J(E_{0})$ are negative, implying that the disease-free equilibrium of the system (1) is locally asymptotically stable. Theorem 1 is proved. □

**Theorem** **2.** *If* $R_{0}<1$*, the disease-free equilibrium of the system (1) is globally asymptotically stable on* Γ.

**Proof.**  If μ≥b, we have

(10) $$\frac{dS_{k}(t)}{dt}\leq b(1-S_{k}(t)-R_{k}(t))-[(1-p)\beta +\gamma p\beta ]k\theta (t)S_{k}(t)-dS_{k}(t)+\mu R_{k}(t).$$

In order to show that the derivative of the Lyapunov function has a non-positive derivative along the system trajectories, the following auxiliary system is constructed

(11) $$\frac{dS_{k}(t)}{dt}=b(1-S_{k}(t)-R_{k}(t))-[(1-p)\beta +\gamma p\beta ]k\theta (t)S_{k}(t)-dS_{k}(t)+\mu R_{k}(t).$$

We present the following Lyapunov function candidate [30]

(12) $$L(t)=\sum_{k}\frac{g(k)}{\lambda +\delta +\gamma }\left(S_{k}(t)-S_{0}-S_{0}\mathrm{In}\frac{S_{k}(t)}{S_{0}}\right)+\sum_{k}\frac{g(k)}{\lambda +\delta +\gamma }I_{k}(t).$$

where $S_{0}=\frac{b}{b+d}$. The derivative of L(t), with respect to t along the solution of system (1), is given by

(13) $$\begin{array}{cc} L^{\prime }(t)= & \sum_{k}a_{k}\left(1-\frac{b}{\left(b+d)S_{k}(t\right)}\right)\cdot {S^{\prime }}_{k}(t)+\sum_{k}a_{k}{I^{\prime }}_{k}(t)\\ = & \sum_{k}a_{k}\left(1-\frac{b}{\left(b+d)S_{k}(t\right)}\right)\cdot [b-(b+d)S_{k}(t)+(\mu -b)R_{k}(t)-[(1-p)\beta +\gamma p\beta ]k\theta (t)S_{k}(t)]+\\ & \sum_{k}a_{k}[[(1-p)\beta +\gamma p\beta ]k\theta (t)S_{k}(t)-(\lambda +\delta +r)I_{k}(t)]\\ \leq & -\sum_{k}a_{k}\frac{{\left[(b+d)S_{k}(t)-b\right]}^{2}}{\left(b+d)S_{k}(t\right)}-\sum_{k}a_{k}\left(\frac{b}{\left(b+d)S_{k}(t\right)}-1\right)(\mu -b)R_{k}(t)+\sum_{k}a_{k}\frac{b}{b+d}[(1-p)\beta +\gamma p\beta ]k\theta (t)-\\ & \sum_{k}a_{k}(\lambda +\delta +r)I_{k}(t)\\ = & -\sum_{k}a_{k}\frac{{\left[(b+d)S_{k}(t)-b\right]}^{2}}{\left(b+d)S_{k}(t\right)}-\sum_{k}a_{k}\left(\frac{b}{\left(b+d)S_{k}(t\right)}-1\right)(\mu -b)R_{k}(t)-(\lambda +\delta +r)(1-R_{0})\theta (t). \end{array}$$

Thus, when μ>b and $R_{0}<1$ for all $R_{k}(t)\geq 0$, we see that $\frac{dL(t)}{dt}\leq 0$ [31].

If μ<b, it follows from (1) that
  (14) $$\frac{dS_{k}(t)}{dt}\leq b-bS_{k}(t)-dS_{k}(t).$$
  For simplicity, we consider the following auxiliary system
  (15) $$\frac{d{\bar{S}}_{k}(t)}{dt}=b-(b+d)S_{k}(t).$$
  There is $S_{k}(t)\leq S_{0}+\varepsilon$ for any ε>0, we have
  (16) $$\frac{dI_{k}(t)}{dt}\leq [(1-p)\beta +\gamma p\beta ]k\theta (t)(S_{0}+\varepsilon )-(\lambda +\delta +r)I_{k}(t).$$
  Similarly, the following auxiliary system is
  (17) $$\frac{dI_{k}(t)}{dt}=[(1-p)\beta +\gamma p\beta ]k\theta (t)(S_{0}+\varepsilon )-(\lambda +\delta +r)I_{k}(t).$$
  In the following, the Lyapunov function candidate is
  (18) $$L(t)=\sum_{k}\frac{g(k)}{\lambda +\delta +\gamma }I_{k}(t).$$
  where $g(k)=\frac{kp(k)}{\langle k\rangle }.$ The derivative of L(t), with respect to t along the solution of system (1), is given by
  (19) $$\begin{array}{cc} \frac{dL(t)}{dt} & =\sum_{k}\frac{g(k)}{\lambda +\delta +\gamma }\left\{\left[(1-p)\beta +\gamma p\beta ]k\theta (t)(S_{0}+\varepsilon )-(\lambda +\delta +r)I_{k}(t\right)\right\}\\ & \leq \sum_{k}\frac{kp(k)}{\langle k\rangle (\lambda +\delta +r)}[(1-p)\beta +\gamma p\beta ]k\theta (t)(S_{0}+\varepsilon )-\sum_{k}\frac{kp(k)}{\langle k\rangle (\lambda +\delta +r)}(\lambda +\delta +r)I_{k}(t)\\ & =\sum_{k}\frac{k^{2}p(k)}{\langle k\rangle }\cdot \frac{\left[(1-p)\beta +\gamma p\beta \right]}{\left(\lambda +\delta +r\right)}\cdot \theta (t)(S_{0}+\varepsilon )-\theta (t)\\ & =\frac{\left[\varepsilon (1-p)\beta +\gamma p\beta \right]}{\left(\lambda +\delta +r\right)}(S_{0}+\varepsilon )\theta (t)\cdot \frac{\langle k^{2}\rangle }{\langle k\rangle }-\theta (t)\\ & =\left\{\frac{\left[(1-p)\beta +\gamma p\beta \right]}{\left(\lambda +\delta +r\right)}(\frac{b}{b+d}+\varepsilon )\frac{\langle k^{2}\rangle }{\langle k\rangle }-1\right\}\theta (t)\\ & =\left\{R_{0}+\frac{[(1-p)\beta +\gamma p\beta ]\varepsilon }{\left(\lambda +\delta +r\right)}\cdot \frac{\langle k^{2}\rangle }{\langle k\rangle }-1\right\}\theta (t). \end{array}$$

Since $R_{0}<1$, the number ε is small enough that $R_{0}+\frac{\varepsilon [(1-p)\beta +\gamma p\beta ]}{\lambda +\delta +r}\leq 1$. Hence $\frac{dL(t)}{dt}\leq 0$ for all $I_{k}(t)\geq 0$, and $\frac{dL(t)}{dt}=0$, if and only if $I_{k}(t)=0$. When t→∞, we have $\underset{t\to \infty }{\lim}I_{k}(t)=0$. According to the above proof, the disease-free equilibrium is globally asymptotically stable. □

**Theorem** **3.** *If* $R_{0}>1$*, the endemic equilibrium of the system (1) is locally asymptotically stable.*

**Proof.**  Let $x_{k}=S_{k}-S_{k}^{*}$, $y_{k}=I_{k}-I_{k}^{*}$, $z_{k}=Q_{k}-Q_{k}^{*}$, $w_{k}=R_{k}-R_{k}^{*}$.

Considering the linear system of the system at point $\left(S_{k}^{*},I_{k}^{*},Q_{k}^{*},R_{k}^{*}\right)$
  (20) $$\left\{ \begin{array}{l} \frac{dx_{k}}{dt}=-\left\{b+d+[(1-p)\beta +\gamma p\beta ]k\theta^{*}\right\}x_{k}+(\mu -b)w_{k}-by_{k}-bz_{k}-[(1-p)\beta +\gamma p\beta ]kS_{k}^{*}\theta ,\\ \frac{dy_{k}}{dt}=[(1-p)\beta +\gamma p\beta ]k\theta^{*}x_{k}+[(1-p)\beta +\gamma p\beta ]kS_{k}^{*}\theta -(\lambda +\delta +r)y_{k},\\ \frac{dz_{k}}{dt}=\delta y_{k}-(r+\eta )z_{k},\\ \frac{dw_{k}}{dt}=\lambda y_{k}+\eta z_{k}-(\mu +d)w_{k}. \end{array}\right.$$
  where $\theta (t)=\sum_{k}\frac{kp(k)}{\langle k\rangle }y_{k}$, $\theta^{*}=\sum_{k}\frac{kp(k)}{\langle k\rangle }I_{k}^{*}$. We obtain
  $$\begin{array}{ll} \frac{d\theta (t)}{dt} & =\sum_{k}\frac{kp(k)}{\langle k\rangle }\{[(1-p)\beta +p\gamma \beta ]k\theta^{*}x_{k}+[(1-p)\beta +\gamma p\beta ]kS_{k}^{*}(\lambda +\delta +\gamma )y_{k}\}\\ & =\frac{[(1-p)\beta +\gamma p\beta ]\theta^{*}}{\langle k\rangle }\sum_{k}k^{2}p(k)x_{k}+\frac{\left[(1-p)\beta +\gamma p\beta \right]}{\langle k\rangle }\sum_{k}k^{2}p(k)x_{k}S_{k}^{*}\theta (t)-(\lambda +\delta +\gamma )\theta (t) \end{array}$$
  According to Equation (7) and further simplification, we can obtain
  (21) $$\frac{d\theta (t)}{dt}=\frac{[(1-p)\beta +\gamma p\beta ]\theta^{*}}{\langle k\rangle }\sum_{k}k^{2}p(k)x_{k}.$$
  In the following, we consider the linear system
  (22) $$\frac{d}{dt}\left(\begin{array}{c} \theta \\ x_{1}(t)\\ y_{1}(t)\\ z_{1}(t)\\ w_{1}(t)\\ \vdots \\ x_{M}(t)\\ y_{M}(t)\\ z_{M}(t)\\ w_{M}(t) \end{array}\right)=A\left(\begin{array}{c} \theta \\ x_{1}(t)\\ y_{1}(t)\\ z_{1}(t)\\ w_{1}(t)\\ \vdots \\ x_{M}(t)\\ y_{M}(t)\\ z_{M}(t)\\ w_{M}(t) \end{array}\right).$$
  Here
  $$A=\left(\begin{array}{cccccc} 0 & C_{1} & \cdots & C_{J} & \cdots & C_{M}\\ D_{1} & A_{1} & \cdots & 0 & \cdots & 0\\ \vdots & \vdots & & \vdots & & \vdots \\ D_{j} & 0 & \cdots & A_{j} & \cdots & 0\\ \vdots & \vdots & & \vdots & & \vdots \\ D_{M} & 0 & \cdots & 0 & \cdots & A_{M} \end{array}\right),C_{i}=\left(\begin{array}{cccc} q_{i}g(i) & 0 & 0 & 0 \end{array}\right),\;D_{i}=(\begin{array}{c} -l_{i}\\ l_{i}\\ 0\\ 0 \end{array})\;\text{and}$$
  $$A_{i}=\left(\begin{array}{cccc} -(b+d+q_{i}) & -b & -b & \mu -b\\ q_{i} & -(\lambda +\delta +r) & 0 & 0\\ 0 & \delta & -(r+\eta ) & 0\\ 0 & \lambda & \eta & -(\mu +d) \end{array}\right).$$
  $$l_{i}=[(1-p)\beta +\gamma p\beta ]iS_{i}^{*},\;q_{i}=[(1-p)+\gamma p\beta ]i\theta^{*}.$$
  The eigenvalues of matrix A are
  $$x_{i1}=-(b+d+q_{i}),\;x_{i2}=-(\lambda +\delta +r),\;x_{i3}=-(r+\eta ),\;x_{i4}=-(\mu +d).$$
  Hence, all the eigenvalues of $J(E^{*})$ are negative. The endemic equilibrium of the system (1) is locally asymptotically stable. Therefore, Theorem 3 is proved. □

## 4. Numerical Simulations

In this section, numerical simulations are given to illustrate the aforementioned theorem results. Firstly, the effect of system parameters on the spreading process is discussed. We take the scale-free network with the degree distribution as $p(k)={\left(\varsigma (3)k^{3}\right)}^{-1}$; the total number of nodes on the network is N=500. The parameters are fixed as following

b=0.4,μ=0.7,d=0.01,λ=0.02,r=0.02,η=0.3.

**Figure 2:**
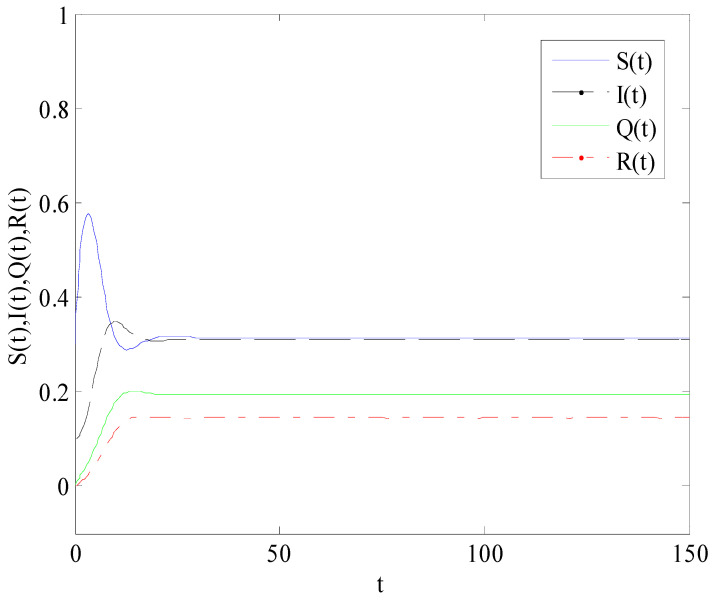
The variation of S(t), I(t), Q(t), R(t) with time when $R_{0}<1$.

**Figure 3:**
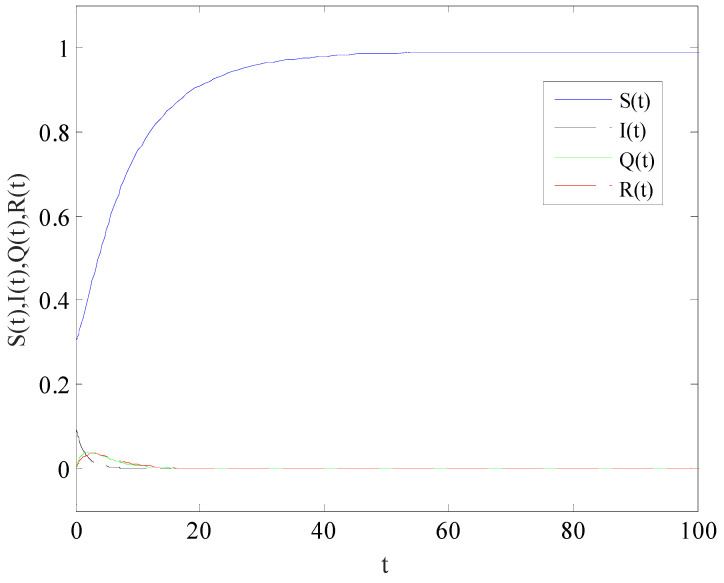
The variation of S(t), I(t), Q(t), R(t) with time, when $R_{0}>1$.

Figure 2 shows the variation of variables of system (1) with time when $R_{0}<1$. There is a globally asymptotically stable disease-free equilibrium point $E_{0}(\frac{b}{b+d},0,0,0,\cdots ,\frac{b}{b+d},0,0,0)$, which implies that the disease is eventually eradicated. Figure 3 gives the variation of variables of system (1) with time when $R_{0}>1$. The system (1) has an endemic equilibrium point $E^{*}$ and reaches the steady state, which will lead to local epidemic disease.

**Figure 4:**
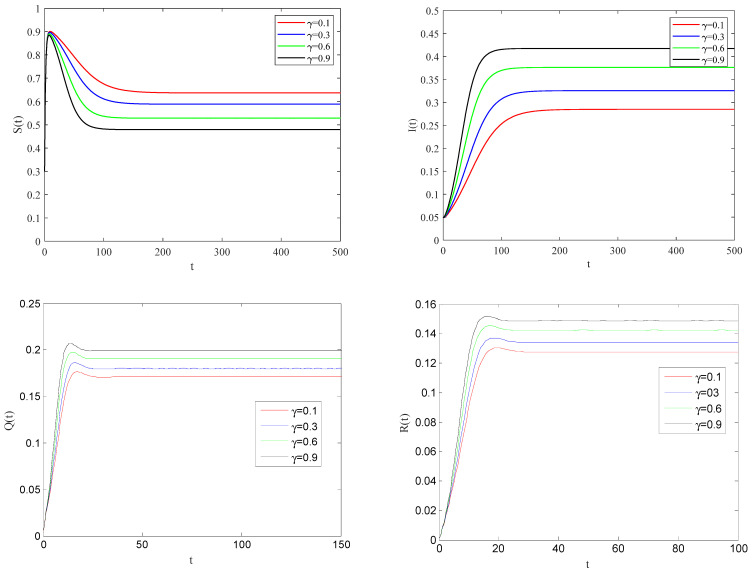
The variation of S(t), I(t), Q(t), R(t) with time for $R_{0}>1$.

Figure 4 shows the impact of the individual alertness probability γ on infectious factor on the spreading process of the epidemic when the basic reproduction number $R_{0}>1$.

From Figure 4, one can observe that the smaller the parameter γ is, the smaller the infection rate of alert individuals will be. This implies that the spreading of infectious diseases becomes slower, and the eventual size of the epidemic will be reduced to a stable state.

**Figure 5:**
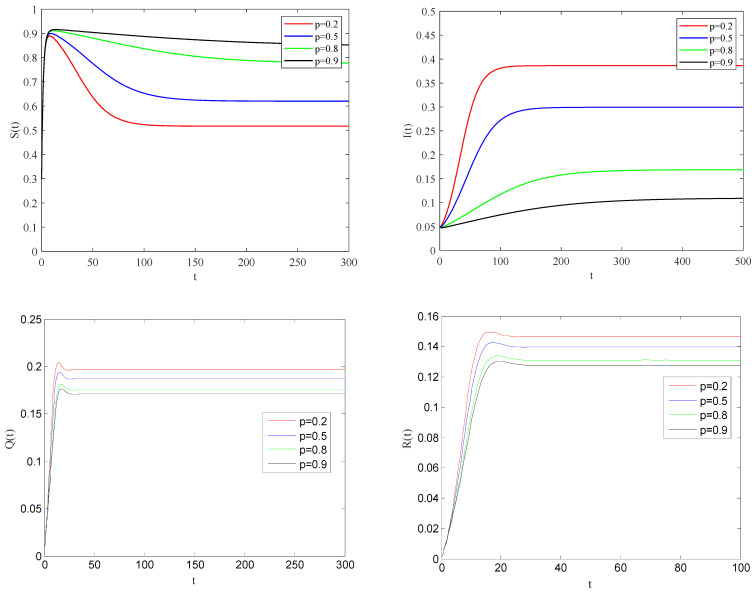
The variation of S(t), I(t), Q(t), R(t) with time for $R_{0}>1$.

Figure 5 illustrates the impact of the individual alertness probability *p* on the spreading process of the epidemic when the basic reproduction number $R_{0}>1$.

**Figure 6:**
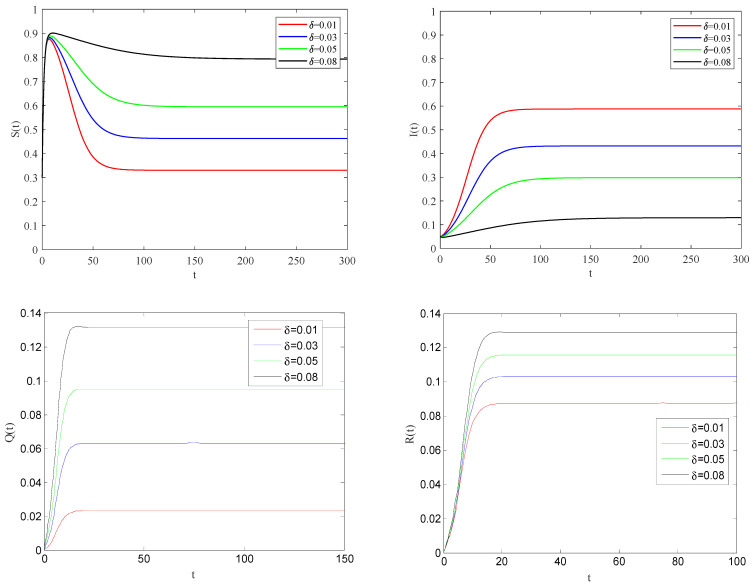
The variation of S(t), I(t), Q(t), R(t) with time for $R_{0}>1$.

One can be see that the larger the alertness rate p, the larger the proportion of individuals taking protective measures will be. Therefore, the probability of contact with infected becomes smaller, which will lead to the decrease in the speed and scale of the spreading process of the epidemic. Figure 6 shows the impact of quarantine rate on the spreading process of the epidemic when the basic reproduction number $R_{0}>1$.

From these figures, it can be observed that the larger the quarantine rate δ, the smaller the rate of infected individuals coming into contact with susceptible individuals. This case means that the infected individuals can be effectively quarantined.

**Figure 7:**
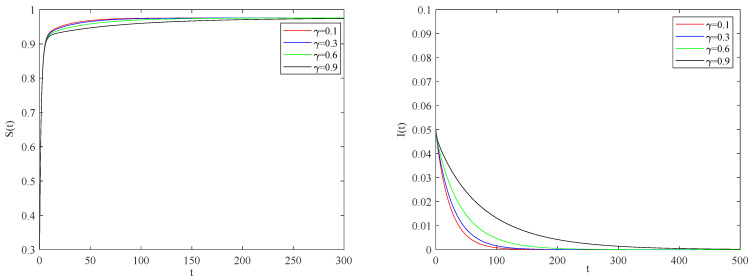
The variation of S(t), I(t) with time for $R_{0}<1$.

Figure 7 presents the impact of individual alertness on infectious factors on the spreading process of the epidemic when the basic reproduction number $R_{0}<1$.

**Figure 8:**
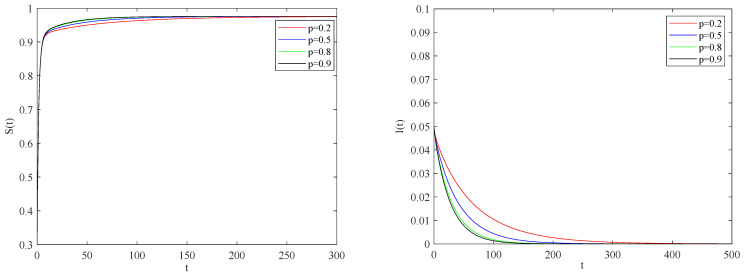
The variation of S(t), I(t) with time for $R_{0}<1$.

We can conclude that the smaller the impact of individual alertness on the infectious factor is, the smaller the infection rate of individuals with an alertness state. Figure 8 describes the impact of individual alertness on the spreading process of the epidemic when the basic reproduction number $R_{0}<1$.

In summary, if the individual alertness rate increases, the probability of susceptible individuals coming into contact with infected individuals will be decreased.

**Figure 9:**
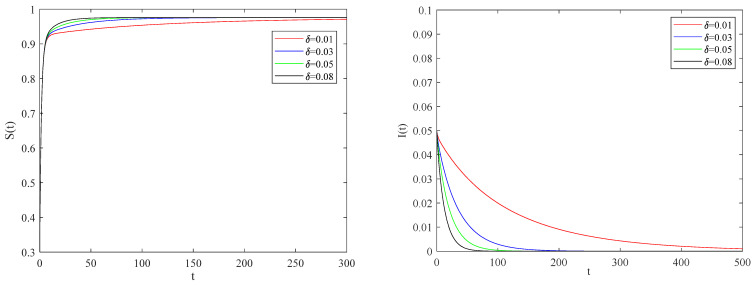
The variation of S(t), I(t) with time for $R_{0}<1$.

Figure 9 indicates the impact of quarantine on the spreading of infection when the basic reproduction number $R_{0}<1$. From these figures, it is found that when the quarantine rate is small enough, an infected individual is not effectively controlled to the extent that the epidemic lasts for a relatively long time.

## 5. Practical Case Simulations

The Coronavirus Disease 2019 is the most dangerous infectious disease for humans in recent years [32]. We study the early spread of COVID-19 in Wuhan in order to describe the importance of individual alertness for infectious diseases. According to the ref. [33], it is known that the number of susceptible people on each grid point at the initial moment is about 1470. Therefore, initial values at the beginning of the disease in Wuhan were S(0)=1470 (data),  I(0)=1 (data), Q(0)=0 (hypothesis), R(0)=0 (data).

**Figure 10:**
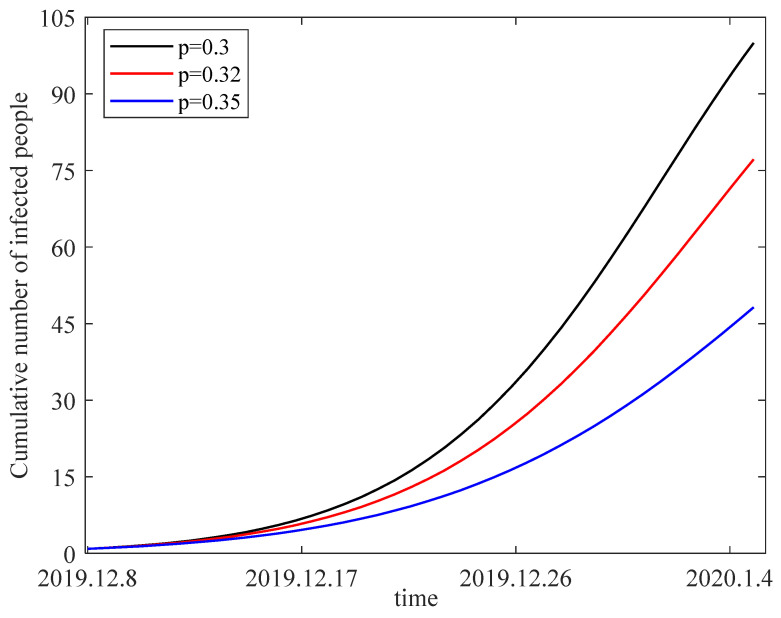
The sensitivity of the number of infected people to the alertness rate.

We fix b=0.06, β=0.1316, γ=0.04, μ=0.9, d=0.01, λ=0.05, δ=0.32, r=0.05, η=0.3. Taking the individual alertness rate p as 0.3, 0.32 and 0.35, we obtain the variation in the number of infected individuals from 8 December 2019 to 5 January 2020 in Figure 10. The figure shows that the number of infected people decreases with an increase in alertness rate. As a result, we should be more vigilant to prevent the transmission of Coronavirus Disease 2019. For example, less frequent visits to public places and frequent disinfection with alcohol are effective measures that can increase individual alertness.

**Figure 11:**
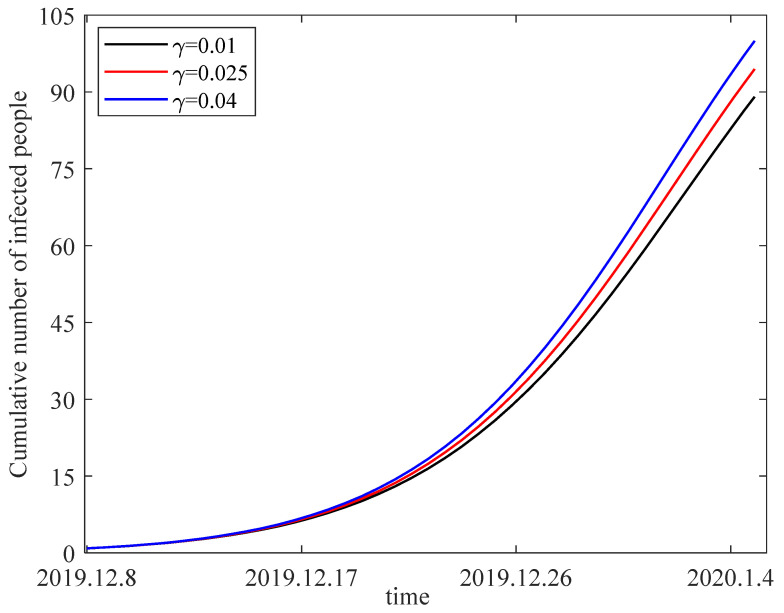
The sensitivity of the number of infected people to individual alertness on infectious factor.

The parameters are selected as b=0.06, β=0.1316, p=0.2, μ=0.9, d=0.01, λ=0.05, δ=0.32, r=0.05, η=0.3. Taking the individual alertness on infectious factors γ as 0.01, 0.025 and 0.04, one can obtain the variation in the number of infected individuals from 8 December 2019 to 5 January 2020 in Figure 11. From the figure, we can generalize that the number of infected people decreases as the impact of individual alertness on infectious factor decreases. The importance that individuals attach to the transmission of infectious diseases also affects the transmission rate of infected individuals.

## 6. Conclusions

This paper presents a new SIQRS network epidemic model with birth and death that considers individual alertness. Individual alertness can lead to a range of protective measures to prevent infection when individuals receive information about the spread of infectious diseases from sources such as the media. Furthermore, the influence of individual behavior on the spread of epidemics is investigated. The basic reproduction number, based on parameters such as birth rate, death rate, individual alertness, individual alertness to infectious factors and quarantine rate, is obtained via the mean field theory. It is further demonstrated that the disease-free equilibrium point is globally asymptotically stable when the basic reproduction number $R_{0}<1$. Meanwhile, if $R_{0}>1$, the intrinsic equilibrium point is locally asymptotically stable. In addition, numerical simulations consider the effect of different values of parameters on the epidemic transmission. Individual alertness can both delay the time when the disease reaches local equilibrium and accelerate the time when it finally dies out. Individual vigilance can both delay the time when the disease reaches local equilibrium and accelerate the time when it finally dies out. Lastly, the intensity of alertness will also determine the size of the endemic disease.

These results imply that individual alertness and quarantine measures play a significant role in the time, speed and scale of epidemic transmission of infectious diseases to reach a steady state, and are important factors in studying the spread and control of infectious diseases. Therefore, the results can provide theoretical guidance for effective prevention and control on epidemic transmission.

## Data Availability

The datasets generated and analyzed during the current study are available from the corresponding author on reasonable request.
